# A multiomics disease progression signature of low-risk ccRCC

**DOI:** 10.1038/s41598-022-17755-2

**Published:** 2022-08-05

**Authors:** Philipp Strauss, Mariell Rivedal, Andreas Scherer, Øystein Eikrem, Sigrid Nakken, Christian Beisland, Leif Bostad, Arnar Flatberg, Eleni Skandalou, Vidar Beisvåg, Jessica Furriol, Hans-Peter Marti

**Affiliations:** 1 Department of Clinical Medicine, University of Bergen, 5021 Bergen, Norway; 2 Spheromics, 81100 Kontiolahti, Finland; 3 Institute for Molecular Medicine Finland (FIMM), University of Helsinki, 00014 Helsinki, Finland; 4 Department of Medicine, Haukeland University Hospital, 5021 Bergen, Norway; 5 Department of Urology, Haukeland University Hospital, 5021 Bergen, Norway; 6 Department of Pathology, Haukeland University Hospital, 5021 Bergen, Norway; 7 Department of Clinical and Molecular Medicine, Norwegian University of Science and Technology, 7491 Trondheim, Norway; 8 St. Olav’s University Hospital, Central Staff, 7006 Trondheim, Norway

**Keywords:** Proteomics, Next-generation sequencing, RNA sequencing, Biomarkers, Renal cell carcinoma

## Abstract

Clear cell renal cell carcinoma (ccRCC) is the most common renal cancer. Identification of ccRCC likely to progress, despite an apparent low risk at the time of surgery, represents a key clinical issue. From a cohort of adult ccRCC patients (n = 443), we selected low-risk tumors progressing within a 5-years average follow-up (progressors: P, n = 8) and non-progressing (NP) tumors (n = 16). Transcriptome sequencing, miRNA sequencing and proteomics were performed on tissues obtained at surgery. We identified 151 proteins, 1167 mRNAs and 63 miRNAs differentially expressed in P compared to NP low-risk tumors. Pathway analysis demonstrated overrepresentation of proteins related to “LXR/RXR and FXR/RXR Activation”, “Acute Phase Response Signaling” in NP compared to P samples. Integrating mRNA, miRNA and proteomic data, we developed a 10-component classifier including two proteins, three genes and five miRNAs, effectively differentiating P and NP ccRCC and capturing underlying biological differences, potentially useful to identify “low-risk” patients requiring closer surveillance and treatment adjustments. Key results were validated by immunohistochemistry, qPCR and data from publicly available databases. Our work suggests that LXR, FXR and macrophage activation pathways could be critically involved in the inhibition of the progression of low-risk ccRCC. Furthermore, a 10-component classifier could support an early identification of apparently low-risk ccRCC patients.

## Introduction

Renal Cell Carcinoma (RCC) constitutes approximately 3% of all cancers worldwide, but its incidence is rising, especially in Western countries^1–3^. Of all subtypes, clear cell Renal Cell Carcinoma (ccRCC) is by far the most common, accounting for approximately 60–70% of all RCC^1^. Localized or low-stage tumors represent its most frequent clinical presentation^4^.

In the last two decades, enormous advances have been made in the development and implementation of medical therapies for ccRCC^5^. However, despite extensive efforts^6,7^, surgery still represents the only curative option, mainly available for localized tumors only^5,7,8^. An improved understanding of the pathophysiology of ccRCC and of their progression is critically required^9–11^ to envisage novel therapeutic approaches to prevent and treat metastatic disease.

Transcriptomics has widely been used to promote the understanding of processes underlying tumor progression. Therefore, the molecular view of ccRCC has mostly been based on gene expression data with inadequate information on protein features^12^. However, the correlation between mRNA and protein levels is far from firm, and quantitative mRNA data alone cannot accurately predict the extent of protein expression associated with ongoing disease processes^13,14^. Thus, there is a need for a more holistic and integrated approach, combining several different omics-related datasets^9–11^. This is particularly important for the identification of subgroups of patients whose clinical outcome is not correctly predictable based on conventional staging/scoring systems. In these cases, a more reliable classification might be of critical relevance for an adequate adjustment of therapeutic protocols.

Predictive classification of ccRCC is usually based on the Leibovich score^15^. However, in a small number of cases, classified as low-risk, tumor progression does occur^16^. These patients might benefit from a more accurate surveillance, from the application of specific adjuvant treatments, and/or from tailored therapeutic regimens.

To address this issue, we have assembled two closely matched cohorts of apparently “low-risk” ccRCC and we have examined the process of disease progression vs. non-progression by integrating three separate levels of -omics data.

Here we show that low-risk progressing ccRCC are characterized by specific molecular features and identify a multiomic signature predicting tumor progression amenable to clinical investigation.

## Materials and methods

### Patients

Tumor tissues were initially collected from a cohort of 443 ccRCC patients treated between 1997 and 2014, in the Haukeland University Hospital (Bergen, Norway). Inclusion criteria were low-risk ccRCC defined by a Leibovich score between 0 and 2, according to the 2003 version of the score. When the updated score was made public in 2018, all cases were re-scored using the updated algorithm^17–20^. No sample lost its status as low-risk in the updated score. In addition to low-risk status, we also required available follow-up data of progression (later occurrence of metastases) or non-progression (absence of tumor recurrence/metastases).

The cohort has been described in detail previously^21^. Briefly, we selected progressors (P) progressing within a 3 months–7 years time range (4.5 years average, n = 8) and, as comparators, two clinically matched non-progressors (NP) per each P (n = 16, 8 years average follow up). A sample was considered matched if the P and NP pair had a similar Leibovich score, age, sex, Fuhrmann grade, tumor stage/size, creatinine levels, and type of surgical tumor removal. Patients, who were not treatment naïve, had lymph node metastasis, suffered from heart failure (grade ≥ 3 according to the New York Heart Association Classification), or used immunosuppressive drugs due to transplantation, or suffered from rheumatic disease at the time of the biopsy were excluded from the study.

All patients showed an estimated glomerular filtration rate (eGFR) > 45 mL/min/1.73 m^2^ at the time of nephrectomy, except for one P with an eGFR of 36 mL/min/1.73m^2^, and a Charlson comorbidity index (CCI) > 1. We examined the patient’s clinical records for information on the follow-up and the development of metastases. Patients’ data are reported on Table 1. NP patients were last evaluated at the end of the follow-up period for this study (11.1.2022), thereby updating the previously published follow-up data^21^.

**Table 1:** Patient characteristics.

Unique ID	Group	Age (year)	Gender	Nephrectomy type	Creatinine	Primary tumour status	Tumour size (mm)	Fuhrmann grade	Leibovich Score 2003^1^	Leibovich Score 2018^2^	Time to metastasis (days)	Pair ID
RCC01	Progressor	72	Female	Radical	106	T1a	20	2	0	2	2680	5
RCC02	Progressor	72	Male	Radical	109	T1b	50	2	2	5	2319	6
RCC03	Progressor	66	Male	Radical	113	T1a	35	3	1	3	2632	3
RCC05	Progressor	83	Male	Radical	81	T1b	50	2	2	5	109	7
RCC06	Progressor	67	Male	Radical	176	T1b	48	2	2	5	1385	8
RCC08	Progressor	63	Male	Radical	60	T1a	40	3	1	3	1994	2
RCC09	Progressor	51	Female	Radical	58	T1a	38	3	2	5	1544	1
RCC11	Progressor	66	Male	Partial	61	T1a	15	3	2	5	965	4
RCC04	Nonprogressor	63	Male	Radical	82	T1b	50	2	2	5		6
RCC07	Nonprogressor	76	Male	Radical	98	T1b	45	2	2	5		7
RCC10	Nonprogressor	78	Female	Partial	64	T1a	20	2	0	2		5
RCC12	Nonprogressor	63	Male	Radical	80	T1b	45	2	2	5		8
RCC13	Nonprogressor	34	Male	Partial	73	T1a	23	3	1	3		1
RCC14	Nonprogressor	72	Male	Partial	97	T1b	55	2	2	5		6
RCC15	Nonprogressor	75	Male	Radical	73	T1b	45	2	2	5		7
RCC16	Nonprogressor	62	Male	Radical	82	T1a	30	3	1	3		3
RCC17	Nonprogressor	54	Male	Partial	68	T1a	35	3	1	3		2
RCC18	Nonprogressor	68	Female	Partial	45	T1a	20	2	0	2		5
RCC19	Nonprogressor	68	Male	Radical	68	T1b	45	2	2	5		8
RCC20	Nonprogressor	66	Male	Radical	67	T1a	30	3	1	3		2
RCC21	Nonprogressor	57	Male	Partial	73	T1a	30	3	1	3		3
RCC22	Nonprogressor	66	Male	Partial	83	T1a	38	3	1	3		4
RCC23	Nonprogressor	74	Male	Partial	81	T1a	16	3	1	3		4
RCC24	Nonprogressor	47	Female	Partial	48	T1a	40	3	1	3		1

One NP patient (RCC19) developed a metastasis after 10 years of follow-up.

**Figure 1:**
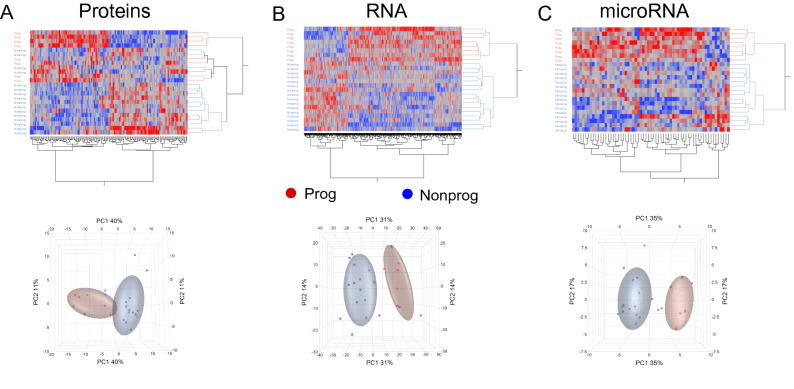
Hierarchical clustering analyses and principal component analyses (PCA). Hierarchical clustering of emerging data results in effective separation of patients’ groups. However, an overlap is still visible in the PCA of the proteomics data (**A**), whereas for mRNA (**B**) and miRNA data (**C**) PCA allows a complete separation of the two groups.

During this process, one patient included in the NP group was revealed to have developed metastasis after a 10-year follow-up. In comparison, the matched progressor sample had developed a metastasis after 3.5 years. The sample was not otherwise clinically distinct from the other NP (Table 1) and did not cluster outside the NP group in systematic expression analysis (Fig. 1). Therefore, considering this very late progression, this patient was still included in the NP group.

The Regional Ethics Committee (REC) of Western Norway approved this study (REC no. 78–05), and all methods were carried out in accordance with relevant guidelines and regulations, including the declaration of Helsinki. Informed consent for their inclusion was obtained from all participants or their legal guardians.

### Tumor specimen collection

Partial or full nephrectomy specimens of all 24 ccRCC patients were sent from the operating room directly to our Department of Pathology for processing and storage as formalin-fixed and paraffin-embedded (FFPE) samples at room temperature. Tissue specimens remaining after routine diagnostic evaluation were used in our study.

### Pathology and staging

As described previously^21^, each sample was initially examined and scored by an experienced renal pathologist (LB) according to Fuhrmann grade. Prior to inclusion in this study each patient was subsequently reassessed and rescored, by the same experienced renal pathologist (LB). The second scoring was performed independently of the first.

### RNA extraction

As described previously^21^, four 10 µm sections per FFPE block were cut for total RNA extraction, which was performed using the miRNeasy FFPE kit (cat no. 217504; Qiagen, Venlo, The Netherlands), as previously described^22,23^. Total RNA concentration was measured using a Qbit RNA HS assay kit on a Qubit 2.0 fluorimeter (Thermo Fisher Scientific, Waltham, MA, USA). RNA integrity was assessed using an Agilent RNA 6000 Nano kit on a 2100 bioanalyser (Agilent Technologies, Santa Clara, CA, USA), and DV200 values (percentage of fragments > 200 nucleotides) were calculated. Following RNA extraction, samples were stored at − 80 °C.

### RNA library preparation and sequencing

As described previously^21^, sequencing libraries were generated using the TruSeq RNA exome library kit (Illumina, San Diego, CA, USA), according to manufacturers’ instructions.

Libraries were quantitated by qPCR using the KAPA library quantification kit–Illumina/ABI Prism (Kapa Biosystems, Wilmington, MA, USA) and validated using the Agilent high-sensitivity DNA kit on a bioanalyzer. Libraries were normalized to 2.6 pM and subjected to cluster and paired-end read sequencing, performed for 2 × 75 cycles on two NextSeq500 HO flowcells (Illumina), according to manufacturers’ instructions. Base-calling was performed using the NextSeq500 instrument, and RTA 2.4.6. FASTQ files were generated using bcl2fastq2 conversion software (v.2.17; Illumina).

### miRNA sequencing

As described previously^24,25^, small RNA sequencing of the 24 samples/libraries was performed using the NEXTflex small RNA-seq kit v3 (Bio Scientific, Austin, TX, USA). The adapter-dimer reduction technology incorporated into this kit allows low input library preparation. Briefly, 100 ng total RNA, extracted from solid tissues, were used as template for 3′ 4 N and 5′ 4 N adenylated adapter ligation, followed by reverse transcription-first strand synthesis. By applying these products as template for second-strand synthesis, double-stranded cDNA was prepared by PCR amplification (22 cycles). Fragments/libraries were run on a Labchip GX (Caliper/PerkinElmer), for quality control and quantitation. Individual libraries were normalized to 25 nM and pooled. The library pool was purified with the QIAquick PCR Purification Kit (Qiagen AB, Sweden), according to providers’ instructions. Automated size selection was performed using the Blue Pippin technology (Sage Science, Beverly, MA, USA), with a range of 135–165 bp to select the ~ 152 bp fragment.

Following size selection, the pool was evaluated on Bioanalyzer (Agilent Technologies, Santa Clara, CA, USA) using the High Sensitivity DNA kit. The pool of libraries was quantified with the KAPA Library Quantification Kit (Roche, Pleasanton, CA, USA). Libraries were quantified by quantitative polymerase chain reaction (qPCR) using the KAPA Library Quantification Kit–Illumina/ABI Prism® (Kapa Biosystems, Wilmington, MA, USA) and validated using the Agilent High Sensitivity DNA Kit on a bioanalyzer. Libraries were normalized to 2.8 pM subjected to clustering. Single read sequencing was performed for 51 cycles on a NextSeq500 HO flowcell (Illumina, San Diego, CA, USA), according to the manufacturer's instructions. Base calling was done on the NextSeq500 instrument by RTA 2.4.6. FASTQ files were generated using bcl2fastq2 Conversion Software v2.17 (Illumina, Inc. San Diego, CA, USA).

### Proteomics sample preparation

For protein extraction, 3–4 tissue sections of 10-μm thickness were used to get approximately equal tissue amounts per specimen. Excess paraffin was trimmed off and sections were deparaffinized using Deparaffinization solution (Qiagen, Cat. No. 19093). Lysis buffer (0.1 M Tris pH 8, 0.1 M dithiothreitol [DTT], 4% sodium dodecyl sulfate) was added to the sections that were homogenized in a Precellys Evolution Homogenizer (BertinPharma, Cat. No. P000062-PEVO0-A) using ceramic (zirconium oxide) beads (BertinPharma, Cat. No. P000933-LYSK0-) and then sonicated. Samples were centrifuged at 15,000*g* for 10 min and supernatants transferred to new tubes. Protein concentration was measured using BCA Protein Assay Kit (Abcam, Cat. No. ab102536) and 20 µg of protein in 20 µL of lysis buffer was used in the following steps.

Samples were reduced using 2 µL of 100 mM Dithiothreitol and alkylated with 3 µL of 200 µM Iodoacetamide. For peptides isolation, SeraMag Speed Beads (GE Healthcare, Cat. No. 45152105050250 and Cat. No. 65152105050250) were used following manufacturer’s instructions. Sequencing grade modified trypsin (0.8 µg per sample) was used to hydrolyse proteins for 16 h at 37 °C. Digested peptides were eluted and desalted using Oasis HLB µElution plates (Waters, Milford, MA), dried in a vacuum centrifuge, and rehydrated in 2% acetonitrile (ACN) and 0.1% formic acid (FA). Peptide concentration was measured using a NanoDrop One (ThermoFisher, Cat. No. ND-ONE-W).

### Liquid chromatography and tandem mass spectroscopy (LC–MS/MS)

As described previously^26^, tryptic peptides, from 0.5 µg of protein dissolved in 2% ACN and 0.1% FA, were injected into an Ultimate 3000 RSLC system (Thermo Scientific) which was connected online to a linear quadrupole ion trap-orbitrap mass spectrometer (Thermo Scientific), equipped with a nanospray Flex ion source (Thermo Scientific). For trapping and desalting, samples were loaded and desalted on a pre-column (Acclaim PepMap 100, 2 cm × 75 µm i.d. nanoViper column, packed with 3 µm C18 beads) at a flow rate of 5 µl/min for 5 min with 0.1% trifluoroacetic acid (vol/vol).

### Computational and Statistical data analysis

#### Proteomics

Raw mass spectrometer files were analyzed using MaxQuant v 1.6.1.0^27^. MS spectra were searched in the Andromeda search engine against the forward and reverse Human Uniprot database (Swissprot reviewed, canonical and isoforms 23.04.18). Label-free quantification was used to identify the relative concentration of proteins in each sample. Proteins with at least two peptide counts were considered to be reliably detected and were included in further analysis. Only proteins with abundance values in seven or more NP samples and four or more P samples were further considered (“quality filtered proteins”). The numbers differ between groups due to different samples abundances (n = 8 and n = 16). Raw data were further processed and statistically analyzed with JMP Genomics (v 9, SAS, North Carolina, USA; www.jmp.com). Raw data were first log2 transformed and any missing data were imputed by multivariate normal imputation.

Multivariate normal imputation, which replaces missing data with predicted values based on the multivariate normal distribution using least squares imputation, was run per sample group using a shrinkage estimator for the covariances, to improve the estimation of the covariance matrix^28^. Data were quantile normalized and standardized, and ANOVA was applied, including groups of matched patients as blocking variable (Table 1, “Pair ID”), in order to preserve the close clinical matching of the P and NP groups. Protein abundance differences between patient groups were considered significant if they reached a minimum fold change of 1.5 and *p-*value ≤ 0.05. Canonical pathways were sorted by the smallest Benjamini–Hochberg adjusted *p-*value. Pathway analysis was performed with Ingenuity Pathway Analysis (v.47547484; Qiagen, Redwood City, CA, USA), with the Ingenuity Knowledge Base used as reference set. The significance values (p-value of overlap) for the canonical pathways are calculated by the right-tailed Fisher's Exact Test. (https://qiagen.secure.force.com/KnowledgeBase/KnowledgeIPAPage?id=kA41i000000L5pACAS).

#### mRNA abundances

FASTQ files were quality controlled with fastqc (v0.11.9) then filtered and trimmed by fastp (v0.20.0). Trimmed sequences were aligned to the genome reference using STAR (v2.7.9a) and quality metrics were extracted with picard CollectRNASeqMetrics (v2.21.5). Transcript counts were generated using quasi alignment (Salmon v1.7.0) to the GRCh38 transcriptome reference sequences^29^.

. An empirical expression filter was applied, including genes with > 1 count per million in at least three samples. Trimmed mean of M values^30^ normalization was applied to adjust for variation in library size. Group was used to determine the difference between the two patient groups, and age matching was accounted for as a blocking factor, with one P and two NP samples per age-matched block. Comparative analysis was performed using the voom/Limma R package (www.Bioconductor.org)^31^.

To reduce unwanted variation induced by unknown sources but avoid overfitting, two surrogate variables were added using the SVA package in R Bioconductor (https://bioconductor.org/packages/release/bioc/html/sva.html). Application of the SVA package in R, indicated that 2 surrogate variables were sufficient to help in this respect. Genes with a *p-*value ≤ 0.05 and an absolute fold change (abs.FC) ≥ 2 were considered differentially expressed. Pathway analysis was performed with Ingenuity Pathway Analysis (v.47547484; Qiagen, Redwood City, CA, USA), with the Ingenuity Knowledge Base used as reference set. The significance values (p-value of overlap) for the canonical pathways were calculated by the right-tailed Fisher’s Exact Test. (https://qiagen.secure.force.com/KnowledgeBase/KnowledgeIPAPage?id=kA41i000000L5pACAS). Canonical pathways were sorted by the smallest Benjamini–Hochberg adjusted *p-*value.

#### microRNA abundances

FASTQ files were quality controlled with fastqc (v0.11.9) then filtered and trimmed by fastp (v0.20.0). Small RNA annotation was performed using the Unitas pipeline v1.7.0^32^. An empirical expression filter was applied, which included genes with ≥ 5 count per million in at least three samples. Trimmed mean of M values^30^ normalization was applied to adjust for variation in library size. Again, group was used to determine the difference between the two patient groups, and age matching was accounted for as a blocking factor, with one P and two NP samples per age-matched block.

Comparative analysis was performed using the voom/Limma R package (www.Bioconductor.org). For microRNA (miRNA) as well, to reduce undesired variation induced by unknown sources while avoiding overfitting, two surrogate variables were added using the SVA package in R Bioconductor (https://bioconductor.org/packages/release/bioc/html/sva.html). Also, for miRNA, genes with a *p-*value ≤ 0.05 and abs.FC ≥ 2 were considered differentially expressed and pathway analysis was performed with Ingenuity Pathway Analysis (v.47547484; Qiagen, Redwood City, CA, USA), with the Ingenuity Knowledge Base used as the reference set. The significance values (p-value of overlap) for the canonical pathways were calculated by the right-tailed Fisher's Exact Test. (https://qiagen.secure.force.com/KnowledgeBase/KnowledgeIPAPage?id=kA41i000000L5pACAS. Canonical pathways were sorted by the smallest Benjamini–Hochberg adjusted *p-*value.

#### Data integration

Multiomics data integration was performed using the R package mixOmics (https://www.bioconductor.org/packages/release/bioc/vignettes/mixOmics/inst/doc/vignette.html) in Bioconductor^33^, and uploading sets of features from the three omics platforms. For the biomarker analysis, a set.seed of 150 was chosen arbitrarily and kept for reproducibility purposes. Clustering was performed with Ward´s method.

### Immunohistochemistry

Immunohistochemistry (IHC) experiments were performed using antibodies against Desmoplakin (DSP) to confirm observations at the proteomic level. We chose DSP due to the high degree of difference (Fold change P/NP; 4.77) found between P and NP samples in the proteomics dataset (see below). IHC was performed on 4-μm-thick FFPE sections, with the following primary antibody: anti-DSP Antibody (1:100, polyclonal, Rabbit, HPA054950, ATLAS ANTIBODIES, Bromma, Sweden) and one hour incubation at pH 6.0. Sections were counterstained with haematoxylin (no. CS70030-2; Dako, Kyoto, Japan).

### qRT-PCR

As described previously^21^, qRT-PCR was performed to confirm AGAP2-AS1 at the mRNA level, as reported previously^21^. We chose AGAP2-AS1 as it was one of the mRNAs that best separated P from NP specimens (see below). qRT-PCR was performed using SuperScript IV VILO master mix with ezDNase (No. 11766050; Thermo Fisher Scientific), TaqMan Fast Advanced master mix (No. 4444556; Thermo Fisher Scientific), and the AGAP2-AS1 primer and probe (Hs01096080_s1, no. 4426961; Thermo Fisher Scientific). qRT-PCR was performed on a StepOne Plus real-time PCR system (Applied Biosystems, Carlsbad, CA, USA), with the gene encoding 40S ribosomal protein S13 (RPS13; Hs01011487_g1, no. 4426961; Thermo Fisher Scientific) used to normalize samples. RNA input for cDNA was 50 ng. We used a no template control as negative control.

Three technical replicates were used to compile an average Ct value, which was used in subsequent analyses. qRT-PCR absolute fold change between groups analyzed by averaging normalized Ct values for each group and determining the ∆∆Ct with averaged values. Significance and *p-*values were evaluated using the *Mann*–*Whitney U* test according to ∆Ct values from each sample.

### Data confirmation

Key findings, based on the molecules making up the classifier and the top 20 features of the proteomics and mRNA datasets were further confirmed by accessing The Cancer Genome Atlas (TCGA) data. We obtained sequencing data related to the identified genes from the GDC TCGA Kidney Clear Cell Carcinoma (KIRC) study, utilizing the Xena Functional Genomics Explorer (https://xenabrowser.net/)^34^. We then examined the prognostic potential for key mRNA findings. The association between molecule expression and prognosis was assessed using the Kaplan Meier method to generate survival plots split between high and low abundance for the molecule being examined. The survival of the different groups was then compared using the log-rank test through the Xena Functional Genomics Explorer. We also examined if the prognostic potential (if any) for each protein/mRNA was present only in low-risk patients or universal for ccRCC overall, irrespective of stage. Proteomic findings were validated using The Human Protein Atlas (http://www.proteinatlas.org), to verify the identified proteins^35^, and prognostic associations were examined and compared to our own results, as described above.

### Ethics approval and consent to participate

The Regional Ethics Committee (REC) of Western Norway approved this study (REC no. 78–05) and permission for their inclusion was obtained from all participants.

## Results

### Patients

Of the 443 ccRCC patients, 8 were both classified as low-risk patients and developed disease progression. These patients were thus included as progressors (P). We also included n = 16 patients classified as low-risk that did not develop disease progression. These patients were included as non-progressors (NP) and were closely matched to the progressive patients, see Materials and Methods. One patient included in the NP group was later revealed to have developed metastasis after a 10-year follow-up. The matched progressor sample had developed a metastasis after 3.5 years and the sample was not clinically distinct from the other NP and did not cluster outside the NP group in systematic expression analysis (Fig. 1).

### Proteomics analysis

Formalin-fixed and paraffin embedded kidney samples obtained at initial surgery from patients with ccRCC with Leibovich scores ≤ 2 were subjected to LC–MS/MS proteomics analysis.

LC–MS/MS identified 28,189 unique peptides mapping to 3,954 proteins. Of these, 3,266 proteins were identified with at least 2 unique peptides (minimum confidence score: 82) and were included in further analyses. The highest number of unique peptides found for a single protein was 178 (Neuroblast differentiation-associated protein AHNAK, UniProt Accession No. Q09666).

To identify proteins with a significantly altered abundance in P compared with NP cancers, we performed ANOVA on 1,220 quality filtered proteins (see “Materials and methods”). Criteria for being considered significantly differentially abundant were set to a *p-*value ≤ 0.05 and an abs. FC ≥ 1.5. A total of 151 proteins met these criteria, of which 75 (49.7%) were more abundant in P than in NP. The twenty proteins with the largest abs. FC are listed in Supplementary document S1.

Expression levels of the 151 differentially abundant proteins did separate the two sample groups when analyzed by unsupervised hierarchical clustering (Fig. 1A, upper panel). However, unsupervised principal component analysis (PCA), used to visualize variance in the data set, indicated that separation of P and NP cancers was not complete, since NP-12 and NP-13 samples were clustering closer to P tissues (Fig. 1A, lower panel). These two samples did not differ from other NP in clinical or technical matters and were therefore not excluded from further analysis.

### mRNA-seq

Statistical analysis (see Materials and Methods) of read counts for 18,942 genes showed that 1167 genes were differentially expressed in P and NP ccRCC with *p-*values ≤ 0.05 and abs. FC ≥ 2. Unsupervised data visualization and dimension reduction techniques revealed that these results effectively separated the two sample groups according to clinical outcome (Fig. 1B upper panel). Importantly, unlike the proteomic data, no overlap of P and NP specimens was detectable upon PCA (Fig. 1A lower panel). The twenty genes with largest abs. FC are listed in Supplementary document S1.

### microRNA-seq

We analyzed sequencing data from 1,894 microRNAs (miRNA). A total of 63 miRNAs passed the pre-set criteria for statistical significance (see above). As shown in Fig. 1C, hierarchical cluster analysis clearly separated the two sample groups, and PCA identified “Diagnosis”, i.e., P vs. NP, as the main source of variance in principal component 1. The twenty miRNAs with largest fold changes are listed in Supplementary Document S1.

### Pathway analysis

**Figure 2:**
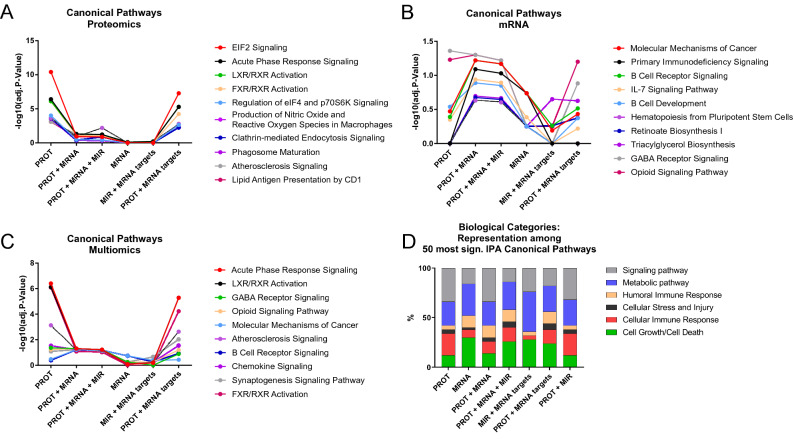
Different omics analyses result in different pathway signatures. Adjusted *p-*values of the 10 pathways with highest significance in the respective analyses were comparatively evaluated. Statistical significance is reached at − log10(adj. *p-*value) > 1.3 (i.e., adj. *p-*value < 0.05). We compared the pathway analysis results of the ten pathways with the lowest adjusted *p-*values from: (**A**) proteomics alone (PROT), (**B**) mRNA-seq alone (MRNA), and (**C**) multiomics analysis (PROT + MRNA + MIR). The large differences in adjusted *p-*value differences between PROT and MRNA in A and B are consistent with very disparate results for their respective pathway signature. (**D**) Percentages of the biological categories attributed to the 50 most significantly affected pathways captured by proteomics, mRNA, and miRNA or their combinations.

Pathway analysis was then used to identify pathway enrichments in the three lists of differentially affected proteins, mRNAs and miRNAs, or their combinations (Table 2 and Fig. 2). The highest number (n = 112) of significantly (*p-*value ≤ 0.05) affected pathways was detected for differentially abundant proteins, whereas the analysis of differentially affected mRNAs yielded 36 significantly affected pathways. In contrast, evaluation of miRNAs expression did not result in the identification of any specific pathway, although the analysis of miRNA-target mRNAs led to the identification of 14 significantly affected pathways (Supplementary Document S2, available at https://figshare.com/articles/online_resource/Untitled_Item/19086512). The twenty most affected pathways identified by proteomic, or mRNA analysis are reported in Table 2, whereas a full list of analyzed pathways, including defined genes and gene products is available in Supplementary Document S2 (https://figshare.com/articles/online_resource/Untitled_Item/19086512). We selected the 10 most significantly affected canonical pathways, as identified in the three analyses with proteomics alone, mRNA-seq alone, and combinatorial multiomics analysis. We then examined how using combinations of the individual omics results as input for the pathway analysis affected the significance levels of those pathways, see Fig. 2A–C, respectively. Most notably, the overlap of the identified pathways was minimal, and a variety of pathways were identified only in the proteomics, or in mRNA-seq data. In particular, proteomic analysis unraveled an overexpression in NP, as compared to P tissues, of proteins associated with translation processes via EIF2 pathway (*p-*value = 1.0E−13), as well as with acute phase response signaling (*p-*value = 2.29E−09), LXR/RXR, FXR/RXR activation (*p-*values = 6.61E−09 and 9.77E−09, respectively), and IL-12 signaling in macrophages (*p-*value = 2.34E−04) (Table 2).

**Table 2:** Twenty pathways from each dataset, sorted by increasing *p-*values.

Ingenuity canonical pathways	P-value	adj. P-value	Ratio	Ratio (# molecules)	P > NP (# proteins)	NP > P (# proteins)
**Proteomics**						
EIF2 signaling	1.00E−13	3.98E−11	0.08	17/224	5	12
Acute phase response signaling	2.29E−09	3.98E−07	0.07	12/180	0	12
LXR/RXR activation	6.61E−09	7.59E−07	0.08	10/121	0	10
FXR/RXR activation	9.77E−09	8.32E−07	0.08	10/126	0	10
Regulation of eIF4 and p70S6K signaling	1.41E−06	9.77E−05	0.05	9/166	3	6
Production of nitric oxide and reactive oxygen species in macrophages	4.17E−06	2.34E−04	0.05	9/189	2	7
Clathrin-mediated endocytosis signaling	4.90E−06	2.40E−04	0.05	9/183	3	6
Phagosome maturation	6.61E−06	2.82E−04	0.05	8/151	6	2
Atherosclerosis signaling	1.95E−05	7.41E−04	0.06	7/127	1	6
Lipid antigen presentation by CD1	2.29E−05	7.76E−04	0.15	4/26	3	1
mTOR signaling	7.08E−05	2.14E−03	0.04	8/210	2	6
CDK5 signaling	7.41E−05	2.14E−03	0.06	6/108	4	2
Antigen presentation pathway	1.17E−04	3.02E−03	0.10	4/39	1	3
Thiosulfate disproportionation III (rhodanese)	1.26E−04	3.02E−03	0.67	2/3	0	2
IL-12 signaling and production in macrophages	2.34E−04	5.37E−03	0.05	6/133	0	6
Virus entry via endocytic pathways	5.37E−04	1.15E−02	0.05	5/102	2	3
Systemic lupus erythematosus signaling	7.59E−04	1.51E−02	0.03	7/229	1	6
Coagulation system	1.51E−03	2.69E−02	0.09	3/35	0	3
Synaptic long term potentiation	1.55E−03	2.69E−02	0.04	5/129	3	2
PPARα/RXRα activation	1.58E−03	2.69E−02	0.03	6/191	2	4

Ingenuity canonical pathways	P-value	adj. P-value	Ratio	Ratio (# molecules)	P > NP (# RNA)	NP > P (# RNA)
**RNAseq**						
Primary immunodeficiency signaling	6.61E−04	1.83E−01	0.16	8/50	2	6
B cell receptor signaling	1.02E−03	1.83E−01	0.09	17/186	9	8
Molecular mechanisms of cancer	1.12E−03	1.83E−01	0.07	29/400	14	15
IL-7 signaling pathway	3.31E−03	4.11E−01	0.16	9/78	2	7
Triacylglycerol biosynthesis	6.92E−03	5.58E−01	0.14	6/44	4	2
Opioid signaling pathway	8.51E−03	5.58E−01	0.07	18/247	13	5
Retinoate biosynthesis I	9.77E−03	5.58E−01	0.15	5/34	3	2
Synaptogenesis signaling pathway	1.12E−02	5.58E−01	0.07	21/312	14	7
Hematopoiesis from pluripotent stem cells	1.17E−02	5.58E−01	0.12	6/49	1	5
GABA receptor signaling	1.20E−02	5.58E−01	0.09	9/95	7	2
B cell development	1.23E−02	5.58E−01	0.14	5/36	3	2
Bladder cancer signaling	1.35E−02	5.61E−01	0.09	9/97	7	2
Cell Cycle: G1/S checkpoint regulation	1.55E−02	5.69E−01	0.10	7/67	3	4
Regulation of the epithelial mesenchymal transition in development pathway	1.70E−02	5.69E−01	0.10	8/84	4	4
Inhibition of matrix metalloproteases	1.74E−02	5.69E−01	0.13	5/39	3	2
PKCθ signaling in T lymphocytes	1.86E−02	5.79E−01	0.08	12/155	8	4
Nicotine degradation III	2.34E−02	6.35E−01	0.10	6/57	5	1
Role of osteoblasts, osteoclasts and chondrocytes in rheumatoid arthritis	2.45E−02	6.35E−01	0.07	15/218	9	6
Oncostatin M signaling	2.51E−02	6.35E−01	0.17	5/43	3	2
Melatonin degradation I	2.95E−02	6.35E−01	0.10	6/60	5	1

Ingenuity canonical pathways	P-value	adj. P-value	Ratio	Ratio (# molecules)	P > NP (# microRNA)	NP > P (# microRNA)
**microRNAseq**						
No pathways detected						

*P* progressor, *NP* nonprogressor.

Instead, transcriptomics analysis, besides a “Molecular mechanisms of cancer” pathway enrichment, revealed a relatively strong representation of pathways related to the adaptive immune system, such as B cell receptor signaling (*p-*value = 1.02E−03) and B cell development (*p-*value = 1.234E−02), cell cycle regulation (*p-*value = 1.55E−02), and, intriguingly, GABA receptor signaling (*p-*value = 1.2E−02).

Differences between each representation of biological processes became even more evident when higher-level biological categories were assigned to the 50 pathways with the lowest adjusted *p-*value from each analysis (Fig. 2D). Indeed, among the 50 most affected pathways in the proteomics analysis, 26% were related to cellular or humoral immune response, and 36% to metabolism. In contrast, in the mRNA-seq-derived list, 62% of the identified pathways were related to metabolism and cell growth, and 20% were associated with immune response. These differences were balanced out when input lists from proteins, mRNAs and miRNAs experiments were combined (Fig. 2D).

### Multiomics integration

To attempt the development of a molecular classifier identifying P ccRCC among low-risk patients and to examine what biological processes might underlie their differential profiles, we integrated the three generated datasets by using mixOmics R package (https://www.bioconductor.org/packages/release/bioc/html/mixOmics.html^36,37^). Its framework DIABLO enables the integration of multiple datasets from the same biological samples for a variety of biological questions. Although some analyses can be performed in a supervised manner, the identification of marker features is eventually achieved in unsupervised steps.

**Figure 3:**
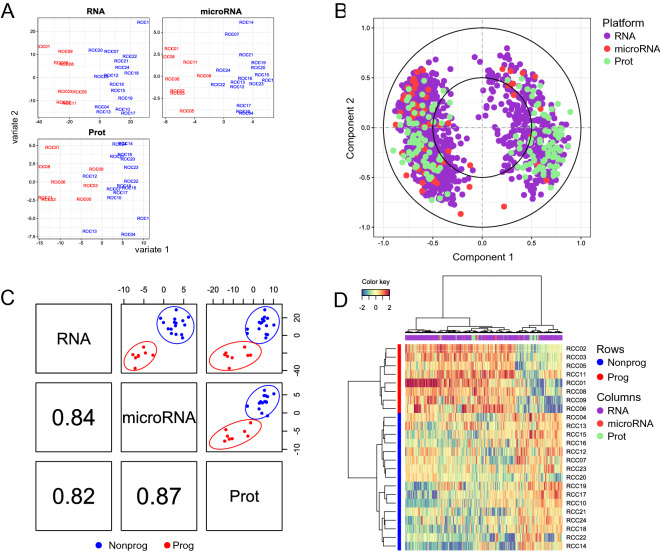
Multiomics integration. Sparse PLS-DA (sPLS-DA) for RNA, miRNA and protein datasets with respective differentially expressed features support the expected separation of sample groups on the first component (**A**). The correlation circle plot (**B**) displays the correlation between variables (biological features) and latent components. Each variable coordinate is defined as the Pearson correlation between the original data and a latent component^58^. The contribution to the definition of each component is visualized as closeness to the circle with radius 1, as well as the correlation structure between variables (clusters of variables). The cosine angle between any two points represents the correlation (negative, positive or null) between two variables. A global overview of the correlation structure for component 1 is shown in (**C**). A strong correlation is detectable for each dataset combination, the strongest being for the combination RNA/microRNA. (**D**) Clustered Image Map (CIM) showing two clusters of samples (rows) and two main clusters of over- and underrepresented features from all three data sources. CIM is based on a hierarchical clustering simultaneously operating on the rows and columns of the selected variables in the original data, here reported by using Euclidian distance and complete linkage.

Following the supervised ANOVA analysis discussed above, partial least square discriminant analysis (PLS-DA) of each individual dataset, shown in Fig. 3A, displayed the separation of samples into two groups, as assigned by P or NP diagnosis, as expected. To visualize the contribution of each variable to each latent component, we used a correlation circle plot (Fig. 3B). All three datasets were highly correlated to each other for component 1, as shown by Pearson correlation plots (Fig. 3C). Moreover, an image map of the multiomics molecular signatures of each sample clearly clustered them according to their diagnosis (vertical axis, Fig. 3D).

Based on the integrated dataset thus generated, we then used the DIABLO-framework to develop a multimodal classifier of early stage ccRCC progression. The tune function was employed to identify a set of features with the best predictive performance. A classifier consisting of 10 components, 3 mRNAs, 5 miRNAs and 2 proteins, evaluated by several rounds of cross-validation, explained the majority of the biological variations underlying the separation into P and NP, and assigned all samples to their respective diagnosis group, with an AUC = 1, an overall error rate (ER) = 0.081, and a balanced error rate (BER) = 0.103.

**Figure 4:**
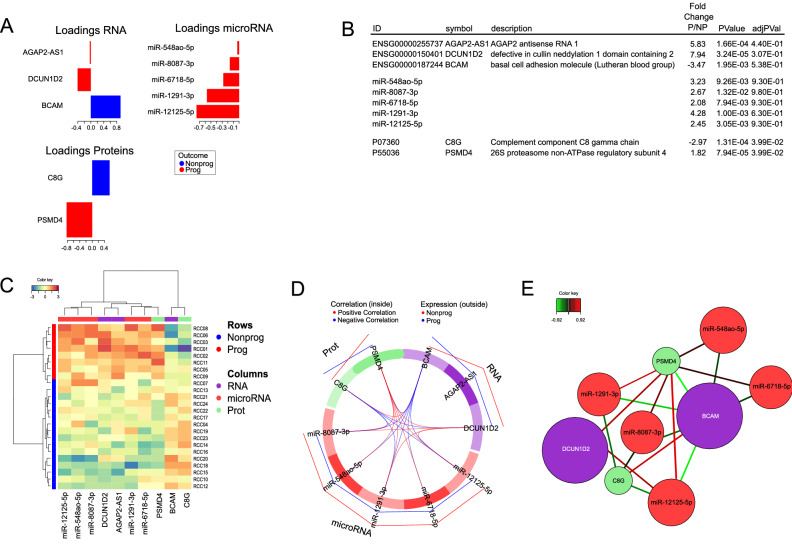
Disease progression signature. For the visualization of the molecular signature, the loading plot (**A**) represents the loading weights of each variable on component 1 of the multivariate model. Most important variables, according to absolute values of their coefficients) are ordered from bottom to top. Colors indicate the class for which the mean expression value is the highest or the lowest for each feature. In (**B**), the features of the model are shown, listed according to the loading plot in (**A**). In (**C**), the Clustered Image Map (CIM) demonstrates that the expression values of the 10 features of the model yield two clusters of samples (rows) and two main clusters of over- and underrepresented features, by employing Euclidian distance and Ward´s linkage. (**D**) Pearson correlation of the expression values of the features, as visualized by a circosplot, with cutoff 0.07. Positive correlation in red, negative in blue. The outer lines indicate whether the featured marker is expressed to a higher (red line) or lower (blue line) extent in NP, as compared to P. The Relevance network in (**E**) demonstrates the correlation structure between variables shown in (**D**) (cutoff 0.7), where positive correlation is depicted in red and negative correlation in blue. The similarity value between a pair of variables is obtained by calculating the sum of the correlations between the original variables and each of the latent components of the model.

The loadings of the set of variables, i.e., the coefficients assigned to each variable to define each component, indicating the importance of each variable in the PLS-DA, are shown in Fig. 4A. A detailed list of the features included in the classifier is provided in Fig. 4B, showing the associated statistical values of the groupwise comparison and the annotation.

Several components of the classifier have previously been associated to prognosis in ccRCC or other cancer types. In particular, the expression of C8G protein is increased in NP low-risk ccRCC. But when examined in ccRCC as a whole, irrespective of cancer stage or risk profile, increased expression is actually associated with poor prognosis in ccRCC (*p-*value = 1.2E−7). ThePSMD4 protein, on the other hand, is overexpressed in P tumors and, accordingly, associated with unfavorable outcome in unselected ccRCC (*p-*value = 1.7E−6) (Table 3). AGAP2*-*AS1 gene expression was increased in P, as compared to NP low-risk ccRCC, and, accordingly, associated with unfavorable prognosis in ccRCC at large (*p-*value = 2.8E−6). Similarly, DCUN1D2 gene expression, enhanced in P “low risk” ccRCC, is also associated with unfavorable outcome in unselected ccRCC. Lastly, BCAM gene expression, increased in NP ccRCC is devoid of prognostic significance in the TCGA KIRC study (*p-*value = 0.409).

**Table 3:** Comparative analysis of the prognostic significance of proteins (A) and genes (B) differentially expressed in P and NP low-risk ccRCC, as detected in this multi-omics evaluation and in unselected ccRCC, based on data from The Cancer Genome Atlas as evaluated in https://www.proteinatlas.org and https://xenabrowser.net, respectively.

	All ccRCC protein overexpression	p	FC P/NP	Low risk ccRCC protein overexpression
**(A)**				
TTR	Unfavorable	0.0082	− 5.09	Favorable
DTD1	Unfavorable	0.0077	− 3.52	Favorable
HMGB1	Irrelevant	0.15	− 3.15	Favorable
TINAGL1	Favorable	0.00041	− 3.01	Favorable
C8G	Unfavorable	1.20E−07	− 2.97	Favorable
SERPING1	Unfavorable	0.006	− 2.85	Favorable
VCAN	Unfavorable	0.00052	− 2.66	Favorable
APOA1	Unfavorable	1.70E−08	− 2.61	Favorable
PLG	Favorable	6.40E−09	− 2.54	Favorable
A1BG	Unfavorable	0.0014	− 2.53	Favorable
HBA1	Favorable	0.0067	− 2.52	Favorable
RPL15	na	na	− 2.42	Favorable
SEPT-2	Unfavorable	0.0096	− 2.4	Favorable
DSP	Favorable	0.00012	4.77	Unfavorable
ITIH1	Unfavorable	0.00077	3.44	Unfavorable
DEFA3	Irrelevant	0.18	3.25	Unfavorable
PTMS	Unfavorable	0.014	2.86	Unfavorable
NPC2	Favorable	0.0031	2.57	Unfavorable
ATP6V1G1	Favorable	3.4E−06	2.49	Unfavorable
PTMA	Unfavorable	0.0016	2.44	Unfavorable

	All ccRCC gene overexpression	p	FC P/NP	Low risk ccRCC gene overexpression
**(B)**				
SLC12A1	Irrelevant	0.343	− 14.31	Favorable
AC146944.1	Irrelevant	0.054	− 11.82	Favorable
IGHA2	Irrelevant	0.142	− 9.18	Favorable
IGLL5	Unfavorable	0.0012	− 9.13	Favorable
SLC9A4	Irrelevant	0.33	− 8.84	Favorable
SV2B	Irrelevant	0.342	14.84	Unfavorable
FAM86B2	Irrelevant	0.52	12.92	Unfavorable
NPIPB9	Unfavorable	0.0048	12.16	Unfavorable
ANKRD20A7P	Irrelevant	0.219	11.74	Unfavorable
LBP	Unfavorable	0.0048	10.86	Unfavorable
ADGRB1	Unfavorable	0.000026	9.56	Unfavorable
ASAH2	Irrelevant	0.292	9.4	Unfavorable
PLK4	Unfavorable	0.0086	9.25	Unfavorable
STAG3	Irrelevant	0.595	9.21	Unfavorable
ZNF321P	na	na	9,09	Unfavorable
AC135048.4	na	na	8.84	Unfavorable
NPIPB1P	Irrelevant	0.06	8.81	Unfavorable
SYS1	Irrelevant	0.831	8.46	Unfavorable
RMRP	na	na	8.18	Unfavorable
SNORD116-18	na	na	10.3	Unfavorable

The favorable and unfavorable designation relates to how the expression of a molecule was associated with a improved of diminished prognosis.

Nevertheless, hierarchical cluster analysis reported in Fig. 4C indicates that classifier variables, including five miRNA markers sufficed to effectively separate P and NP ccRCC. Pearson correlations, reported as circle Plot in Fig. 4D showed that some components of the classifier were characterized by strongly correlated or anti-correlated expression profiles. The relevance network reported in Fig. 4E visualizes the expression correlations from Fig. 4D with a r = 0.7 threshold which can help in the biological interpretation of the results. For instance, these data suggest an anti-correlation of mir-1291 and C8G, consistent with C8G gene being a target of miR-1291.

### Confirmation in external databases

Identified protein and gene markers appeared to enable effective classification of P and NP tumors among low-risk ccRCC, while also reflecting the underlying pathobiology. However, we were also interested in their clinical and prognostic significance in ccRCC at large, irrespective of putative recurrence risk. The expression of the proteome and mRNA markers of prognostic relevance in putatively low-risk ccRCC (Table 3), was detectable at the protein and gene level in unselected ccRCC from publicly available databases (https://www.proteinatlas.org^38^; (https://xenabrowser.net/^34^).

For a number of markers, in these databases, protein and gene expression levels were of concordant prognostic significance. Discordant results were found for others, such as VCAN. Overall, for the top 20 mRNA features, see supplementary document S1, 17/20 features exhibited the same prognostic association both for the mRNA of the gene and the protein from the same gene, e.g., if a high expression of the mRNA meant a favorable prognosis then a high expression of the protein also meant a favorable prognosis. For the top 20 protein features 11/20 features exhibited the same prognostic associations (Supplementary Document S3, available at https://figshare.com/articles/online_resource/Untitled_Item/19086512).

Prognostic significance of the expression levels of a variety of proteome and mRNA markers in whole ccRCC cohorts and in putatively low-risk ccRCC was then explored (Table 3). As an example, overexpression of TINAGL1 protein, detected to higher extents in NP cancers, was also associated with improved prognosis in unselected ccRCC cohorts (*p-*value = 0.00041). Interestingly, however, in other cases, the prognostic significance of the expression levels of a variety of protein markers in unselected ccRCC cohorts and in putatively low-risk ccRCC was markedly discordant. For instance, APOA1 and C8G protein expression was associated with poor prognosis in unselected ccRCC tumors, but detectable at higher levels in our NP than P low-risk tumors. These data support the presence of a high degree of clinical and biological specificity of well-defined ccRCC cohorts, though further examination in other narrowly defined groups is needed.

Regarding mRNA markers, TCGA data (https://xenabrowser.net) indicated that overexpression of ADGRB1 gene, detected in P, as compared with NP tumors, was accordingly associated with decreased survival (*p-*value = 0.000026), in unselected ccRCC. Similar results were found for HBA1 and PLG, see Table 3 for the full results. However, not all results were concurring. A full list of mRNA markers and their expression in the TCGA dataset is provided in Table 3.

### Validation of selected gene and protein signatures with qRT-PCR and IHC

**Figure 5:**
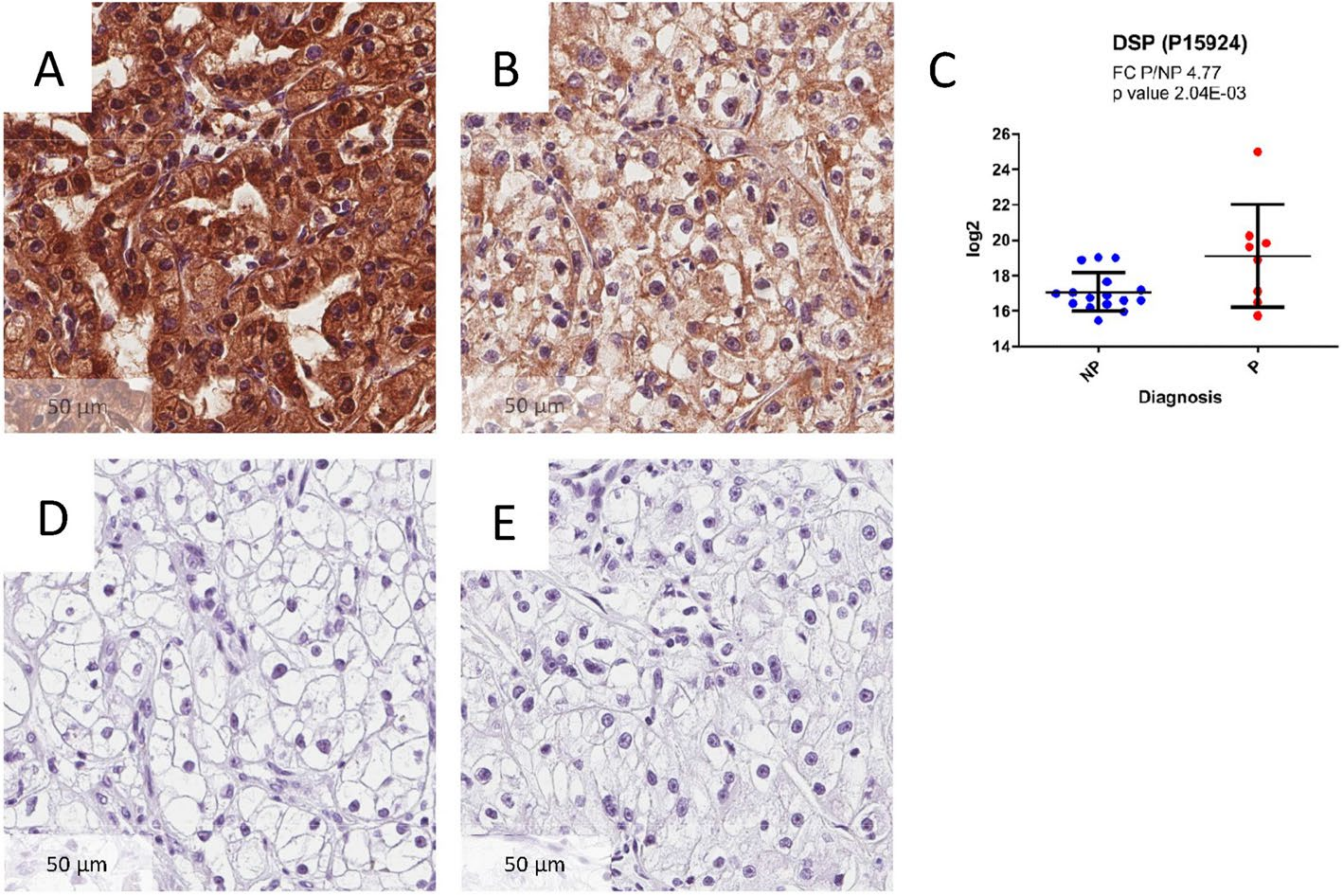
Immunohistochemistry (IHC) of Desmoplakin (DSP) and its protein abundance plot. DSP was expressed to a higher extend in P (**A**), compared to NP (**B**) tumors based on both proteomics data and IHC results. IHC results (40 ×) are from a matched sample pair. The image was taken from the most markedly stained sections of each slide. (**C**) depicts the log2 abundance values of DSP in the proteomics dataset. (**D**) Depicts the staining without the primary antibody. (**E**) Depicts the staining without the secondary antibody.

To validate selected findings, we performed immunohistochemistry for Desmoplakin (DSP), and qRT-PCR for AGAP2-AS1. DSP showed a higher abundance in P compared to NP tumors (Fig. 5A,B). These data were consistent with the proteomic dataset (FC P/NP 4.77, *p-*value = 2.04E−03) (Fig. 5C). Control images with omitted primary (Fig. 5D) and secondary antibody (Fig. 5E) are also provided. In addition, AGAP2-AS1 gene was found to be overexpressed in P tumors also by RT-qPCR (p = 0.035, FC (P/NP): 4.09), as previously reported^21^.

## Discussion

Early detection of susceptibility to cancer progression is essential for treatment and surveillance adjustment. Currently, there is no biomarker indicating if a “low-risk” ccRCC patient will eventually progress towards a higher-grade cancer. To fill this knowledge gap, we have investigated molecular profiles of ccRCC tissues from patients conventionally classified as low-risk, some of which remained progression-free over 10 years postoperatively, whereas others progressed to tumor recurrence. Our investigation resulted in a ten-component classifier of low-risk ccRCC, correctly predicting the progression of low-risk ccRCC years in advance of the advent of progression.

We have generated a multiomics dataset of mass spectroscopy proteomics, RNA-seq and miRNA-seq from renal biopsies of clinically closely matched NP and P patients. Each of these datasets displays specific characteristics, as indicated by the very different nature of differentially expressed markers, and the profiles of apparently involved pathways. This was at least in part expectable, possibly due to the presence in our biopsies of soluble proteins or receptor ligands, produced in non-malignant tissues, outside tumor location. Moreover, defined ccRCC infiltrating cell types, e.g., myeloid cells, are typically characterized by low transcriptional activity^39–41^. A similar discrepancy has also been previously observed by others^42^.

Importantly, key proteins and mRNA abundances in our cohort matched with TCGA data^34^, such as TINAGL1, PLG or HBA1 proteins in NP tumors was accordingly associated with improved prognosis in unselected ccRCC. Similarly, overexpression of ITIH1, PTMS and PTMA proteins in P tumors was consistent with their associated poor prognosis significance in TCGA data from unselected ccRCC, e.g. not selected for stage or risk profile. The components of the classifier have also previously been connected to a variety of malignancies (Supplementary document S4).

The prognostic significance of a number of proteins differentially detectable in P compared to NP tumors did not fully match that observed in unselected ccRCC, as reported in publicly available databases. Examples include TTR and DTD1, unfavorable in unselected but favorable in low-risk ccRCC, or DSP and ATP6V1G1, favorable in unselected but unfavorable in low-risk ccRCC. A similar pattern of discordant prognostic significance was also detectable in the analysis of transcriptomic data. These data suggest that the clinical relevance of the expression of defined markers could be, at least in part, stage/score specific. In this context it is nevertheless remarkable that the use of recombinant TINAGL1 has recently been proposed for cancer treatment^43^.

Our findings support a significant discrepancy both between different cancer stages, but also between proteomics and transcriptomics data, as there were significant gaps in the overlap between expressed mRNAs and proteins. This may suggest that proteins produced outside tumor location may significantly impact on unselected ccRCC clinical outcome. Alternatively, discrepancies might be due to long protein half-lives.

Although proteomics data did not fully separate P and NP tumors, they were characterized by a higher statistical significance, as compared to mRNA or miRNA data, a feature that has been observed previously in multiomics papers^42^.

In particular, “LXR/RXR and FXR/RXR Activation”, “Acute Phase Response Signaling”, “IL-12 signaling and production in macrophages” and “Antigen presentation” pathways were enriched in the proteomics dataset of NP compared to P samples.

. Interestingly, LXR agonists have been shown to inhibit the proliferation of renal cancer cells^45^ and steer macrophage polarization towards an anti-inflammatory profile^46^. In renal cancers at large, acute phase reactants predict poor survival, although less accurately than clinical stage^51^, and C-reactive protein (CRP), represents a clinically significant unfavorable prognostic factor in a variety of urological cancers^52–54^. Importantly, by integrating HLA ligand omics, transcriptomics, genetic and epigenetic data, candidate ccRCC antigens have successfully been identified^55^. Therefore our data consistently support an important role of LXR and of macrophages in the inhibition of ccRCC progression and warrant additional experimental studies to clarify involved molecular mechanisms.

Pathways associated with mRNA data were also identified but they were of minor statistical significance. Expectably, “Molecular mechanisms of cancer” pathway genes were overrepresented in P tumors. Most interestingly, however, a concordant enrichment of “B cell receptor signaling”, “B cell development” and “GABA Receptor Signaling” pathways was observed in P low-risk ccRCC. Indeed, B cells have most recently been reported to produce GABA^56^, which, in turn, promotes macrophage polarization towards an immunosuppressive functional profile. Taken together, these data might suggest that conditioning of the immune system, and, in particular, of macrophage activation, might play a major role in the clinical outcome of low-risk ccRCC.

The absence of a sufficient number of publicly available datasets comparing ccRCC P to NP, particularly in putatively low-risk tumors, and not just “ccRCC” to “healthy” tissue, underlines a need for specific studies like the one presented here. Most interestingly, despite a lack of clinical or macroscopic differences between the patient groups included in our analysis, we unraveled specificities, at the molecular level, mirroring, in part, published tumor biology advances. Notably we report here only one out of many possible panel classifiers, based on its relatively low number of components, rendering it more easily amenable to a clinical application. Remarkably, distinct components of this classifier were connected to each other. As an example, miR-1291 has previously been shown to target C8G^57^, and the two markers are strongly negatively correlated in our dataset.

The most important limitation of our study is represented by the small sample size, although we have included all available patients from our institutions over 17 years. However, low-risk ccRCC progressors are exceedingly rare, constituting only approximately 1.8% of the patient population and requiring almost a decade of follow-up for their identification^16^.

Another potential limitation is represented by a large use of un-adjusted *p-*values, since analysis of -omics data usually requires statistical adjustments of baseline *p-*values to account for multiple testing and false positive results. In our case, due to the close matching of the samples, such adjustments were impossible, and resulted in a loss of significance, with the exception of canonical pathways enrichment in proteomics data, even though the results were validated externally or with other methods, thus supporting a real biological difference between P and NP ccRCC. Moreover, similar limitations have been reported previously in closely matched cohorts^16^. In order to account for this, we have performed several validation experiments and analyses on the TCGA data, all of which accounted for multiple testing when applicable.

Nevertheless, our study also has several strengths mostly based on the close matching of included patients. For each P, we included two matched NP with similar Leibovich score, age, sex, Fuhrmann grade, tumor stage and -size, similar creatinine levels and similarly performed surgical removal of tumors. The thorough matching of subjects reduced unwanted biological and demographic variations, e.g., age differences between the groups, which might have otherwise introduced important bias. Therefore, our findings provide new insights into the progression of putatively low-risk ccRCC and could contribute, as baseline data, to future studies further validating the model, and potentially leading to adjusted treatments for low-risk progressors, or to development of novel therapeutic regimens.

## Conclusion

A combination of omics datasets can be useful for the identification of pathways and molecular signatures associated with progression of low-risk ccRCC. Our work suggests that LXR, FXR and macrophage activation pathways could be critically involved in the inhibition of the progression of low-risk ccRCC. Furthermore, a 10-component classifier could support an early identification of apparently low-risk ccRCC patients more likely to show disease progression, and thus assist in earlier treatment adjustments.

## Supplementary Information

Supplementary Information 1.

Supplementary Information 2.

Supplementary Information 3.

Supplementary Information 4.

## Data Availability

The mRNA data is available through the GEO repository, accession number GSE171955, as is the miRNA data, accession number GSE207557. The proteomics data are available through the GITHUB repository, under the title ‘’A-multiomics-disease-progression-signature-of-low-risk-ccRCC’’. Supplemental data is available through figshare; https://figshare.com/articles/online_resource/Untitled_Item/19086512.
